# Complement peptide C3a receptor 1 promotes optic nerve degeneration in DBA/2J mice

**DOI:** 10.1186/s12974-020-02011-z

**Published:** 2020-11-11

**Authors:** Jeffrey M. Harder, Pete A. Williams, Catherine E. Braine, Hongtian S. Yang, Jocelyn M. Thomas, Nicole E. Foxworth, Simon W. M. John, Gareth R. Howell

**Affiliations:** 1 The Jackson Laboratory, Bar Harbor, ME USA; 2 Division of Eye and Vision, Department of Clinical Neuroscience, St. Erik Eye Hospital, Karolinska Institutet, Stockholm, Sweden; 3 Zuckerman Mind Brain Behavior Institute, New York, NY USA; 4 Department of Ophthalmology, Tufts University of Medicine, Boston, MA USA; 5 Howard Hughes Medical Institute, Department of Ophthalmology, Columbia University Medical Center, and Zuckerman Mind Brain Behavior Institute, New York, NY USA; 6 Sackler School of Graduate Biomedical Sciences, Tufts University School of Medicine, Boston, MA USA; 7 Graduate School of Biomedical Sciences and Engineering, University of Maine, Orono, ME USA

**Keywords:** Glaucoma, Complement, Anaphylatoxin, Microglia, Monocytes, Neurodegeneration

## Abstract

**Background:**

The risk of glaucoma increases significantly with age and exposure to elevated intraocular pressure, two factors linked with neuroinflammation. The complement cascade is a complex immune process with many bioactive end-products, including mediators of inflammation. Complement cascade activation has been shown in glaucoma patients and models of glaucoma. However, the function of complement-mediated inflammation in glaucoma is largely untested. Here, the complement peptide C3a receptor 1 was genetically disrupted in DBA/2J mice, an ocular hypertensive model of glaucoma, to test its contribution to neurodegeneration.

**Methods:**

A null allele of *C3ar1* was backcrossed into DBA/2J mice. Development of iris disease, ocular hypertension, optic nerve degeneration, retinal ganglion cell activity, loss of RGCs, and myeloid cell infiltration in *C3ar1*-deficient and sufficient DBA/2J mice were compared across multiple ages. RNA sequencing was performed on microglia from primary culture to determine global effects of *C3ar1* on microglia gene expression.

**Results:**

Deficiency in *C3ar1* lowered the risk of degeneration in ocular hypertensive mice without affecting intraocular pressure elevation at 10.5 months of age. Differences were found in the percentage of mice affected, but not in individual characteristics of disease progression. The protective effect of *C3ar1* deficiency was then overcome by additional aging and ocular hypertensive injury. Microglia and other myeloid-derived cells were the primary cells identified that express *C3ar1*. In the absence of *C3ar1*, microglial expression of genes associated with neuroinflammation and other immune functions were differentially expressed compared to WT. A network analysis of these data suggested that the IL10 signaling pathway is a major interaction partner of C3AR1 signaling in microglia.

**Conclusions:**

C3AR1 was identified as a damaging neuroinflammatory factor. These data help suggest complement activation causes glaucomatous neurodegeneration through multiple mechanisms, including inflammation. Microglia and infiltrating myeloid cells expressed high levels of *C3ar1* and are the primary candidates to mediate its effects. C3AR1 appeared to be a major regulator of microglia reactivity and neuroinflammatory function due to its interaction with IL10 signaling and other immune related pathways. Targeting myeloid-derived cells and C3AR1 signaling with therapies is expected to add to or improve neuroprotective therapeutic strategies.

**Supplementary Information:**

The online version contains supplementary material available at 10.1186/s12974-020-02011-z.

## Introduction

Glaucoma is a common disease that damages the optic nerve and impairs vision [1]. Risk for glaucoma is greatly increased after middle age and by exposure to elevated intraocular pressure (IOP). Elevated IOP and aging are associated with neuroinflammation, yet it remains unclear when and how neuroinflammation becomes damaging in glaucoma and how to intervene [2, 3]. These questions underlie a need to develop a comprehensive understanding of inflammatory processes in glaucoma.

A major type of inflammatory response observed in glaucoma patients is activation of the complement cascade [4–6]. The complement cascade is activated by three distinct pathways, the classical, alternative, and mannose-binding lectin pathways, which play a key role in responding to tissue damage and infection. The final product of the complement cascade, the membrane attack complex (MAC), has been identified in optic nerve tissue from ocular hypertensive patients. This suggests full activation of the complement cascade has occurred, including multiple steps that promote neuroinflammation. The major products of the complement cascade that regulate neuroinflammation are complement activation peptides and the MAC [7, 8]. The two primary complement activation peptides are polypeptides produced by the cleavage of complement components 3 and 5, and named C3a and C5a. C3a and C5a bind to different cell surface G protein coupled receptors, C3AR1 and C5AR1, respectively. Both receptors can be expressed by glia, neurons, and infiltrating immune cells in the central nervous system. However, whereas C5AR1 largely promotes activation of immune cells, the outcome promoted by C3AR1 varies by the type of injury, cell, and costimulation involved in the inflammatory response [9]. The MAC is a complex formed on plasma membranes by complement components 5b, 6, 7, 8, and 9 as a result of opsonized antigens. Low levels of the MAC on target cells activate intracellular signaling pathways and high levels induce lysis. Sublytic levels of the MAC amplify inflammatory intracellular signaling pathways by activating the NFκB signaling and inflammasome pathways [10, 11]. Due to the potentially damaging role of inflammation in glaucoma and other neurodegenerative disorders, the complement activation peptides and the MAC are predicted to be useful targets for developing anti-inflammatory therapies [12, 13].

Research in animal models suggests that the complement cascade contributes to pathology in ocular hypertensive eyes [4, 14–21]. This includes models of glaucoma like DBA/2J mice, who develop an ocular hypertensive disease in which the complement component 1q complex (C1q) or C5 exacerbates neuroinflammation, retinal ganglion cell loss and optic nerve degeneration [21–24]. These data further support the need to determine the function of pro-inflammatory products of the complement cascade after an ocular hypertensive insult. To test the function of complement activation peptide C3a in a chronic, age-related model of glaucoma, we backcrossed a null allele of the C3a receptor (*C3ar1*^*-*^) into DBA/2J mice. C3AR1 is a G-protein coupled receptor expressed in cells in the nervous and immune systems (see review: [25]) and is implicated in neuropathology in several diseases [26–30]. In DBA/2J mice, *C3ar1* deficiency decreased the incidence of optic nerve damage and RGC loss at a time point consistent with C3a promoting neurodegeneration.

## Methods

### Animals and husbandry

C.129S4-*C3ar1*^*tm1Cge*^/J (*C3ar1*^−^) mice were obtained from The Jackson Laboratory (Bar Harbor, ME, USA; stock number 005712) [31]. The *C3ar1* null allele was backcrossed onto DBA/2J (D2) for 10 generations to generate the congenic strain D2.129S4(C)-*C3ar1*^*tm1Cge*^/Sj. Experimental cohorts of mice were produced by intercrossing heterozygous (*C3ar1*^*+/*−^) mice. Mice of both sexes were used, with approximately equal numbers for each age group and genotype. Mice were housed with a 14-h-light/10-h-dark cycle as previously described [32]. All animals were treated according to the guidelines of the Association for Research in Vision and Ophthalmology for use of animals in research. The Animal Care and Use Committee of The Jackson Laboratory approved all experimental procedures.

### Clinical assessment

Assessment of iris disease was performed using a slit-lamp biomicroscope as previously reported [33] and mice were assessed every 2 months beginning at 6 months of age. IOP was measured by the microneedle method while mice were under anesthesia (ketamine/xylazine) [34, 35]. Mice were assessed every 2 months beginning at 8 months of age. Iris disease and IOP data were collected for at least 40 eyes of each age and genotype.

### Optic nerve damage

Damaged axons stain darkly when treated with the sensitive chemical marker paraphenylenediamine (PPD) [36]. We assessed optic nerve damage by staining cross-sections of the retro-orbital optic nerve with PPD. Two masked investigators assigned each optic nerve one of three damage levels: no or early (NOE; no readily detectible axon loss), moderate (MOD; less than 50% of axons damaged/lost), and severe (SEV; more than 50% of axons damaged/lost). This method of evaluating optic nerve damage has been carefully validated by counting axons [21, 37–40]. Glaucomatous axon damage was assessed in 10.5- and 12-month-old *C3ar1*^*+/+*^ and *C3ar1*^−/−^ mice (55 nerves for each age and genotype).

### RGC soma loss

Eyes were fixed overnight in 4% paraformaldehyde. Retinas were dissected, flat-mounted, and Nissl-stained with cresyl violet as previously described [39]. Images of 40× fields of the RGC layer were obtained using a Zeiss AxioImager. To account for regional variation in RGC density, two 40× fields were counted in each retinal quadrant equidistant to the periphery. The counts in the eight fields were averaged to obtain a single count per eye. Eight eyes were counted per optic nerve damage level and genotype. It is important to note that the RGC layer consists of roughly 50% RGCs. This limits the extent of total neuron loss measured because only RGCs die in standard DBA/2J mice. Loss of RGCs by Nissl staining correlates well with loss of RGCs by axon count in optic nerves with severe damage [21, 37–40].

### Pattern electroretinography

PERG was performed as previously described [41]. Briefly, mice were anaesthetized using ketamine/xylazine [35] and their body temperature was maintained at 37 °C. Eyes were stimulated asynchronously by contrast-reversal of gratings (0.05 cycles/degree, 100% contrast) generated on LED tablets. PERG signals were acquired using subcutaneous needles placed in the snout. Waveforms were determined using the average of three consecutive repetitions.

### RNA isolation from cultured microglia

Primary mixed cortical cultures of glial cells from 3-day-old pups were generated and microglia were fluorescently labeled and sorted as previously described [18]. In brief, 17 days after plating, cultures were dissociated (HyClone Trypsin .25%; Thermo Scientific) and resuspended in FACS buffer: HBSS (Gibco; Invitrogen 14025) supplemented with 2% BSA (Sigma-Aldrich, A7906) and containing 1 U/μl SUPERase In™ RNase Inhibitor (Ambion; Life Technologies, AM2694). Cells were centrifuged at 1305 g for 5 min and suspended in 50 μl of fresh FACS buffer to wash. The cells were stained for 1 h at 4 °C with chicken anti-GFAP (Abcam, ab4674) to label astrocytes and rabbit anti-IBA1 (Wako, 016-20001) to label microglial cells. Cells were washed three times and incubated for 30 min at 4 °C with secondary antibodies: donkey anti-rabbit 647 (Invitrogen, A31573) and goat anti-chicken 488 (Invitrogen, A11039). Samples were re-suspended in 200 μl of FACS buffer and sorted on BD Biosciences LSR II SORP. Purified microglia were collected separately and stored in RLT Buffer (QIAGEN, 79216) at − 80 °C. Total RNA was isolated (QIAGEN, 74104) from purified samples from D2.*C3ar1*^−/−^ and D2.*C3ar1*^*+/+*^ mice.

### RNA-sequencing and analysis of differentially expressed genes

The steps taken to produce sequencing libraries have been previously reported [18]. In brief, starting with 5 ng of high-quality RNA, sequencing libraries were constructed using Ovation RNA-Seq V2 and TruSeq DNA sample prep kit v2 kits. Libraries were sequenced on a HiSeq 2000 sequencer from Illumina. Reads with 70% of their bases having a base quality score ≥ 30 were retained for further analysis. Read alignment and expression estimation were performed using TopHat v 2.0.7 [42] and HTSeq [43] with default parameters against mouse genome (build-mm10). Differentially expressed (DE) genes between groups were identified using edgeR (v 3.8.5) [44] following the removal of lowly expressed genes (counts per million < 1 in more than two samples). The DE gene set was analyzed using ingenuity pathway analysis (IPA) software. Results for enrichment of IPA canonical pathways and upstream regulator terms are shown.

### Myeloid-derived cell counting by flow cytometry

Mice were euthanized and eyes were immediately enucleated. Retinas, optic nerves, and spleens were dissected in ice-cold, filter sterilized HBSS (Gibco; 14175-095) and placed in HBSS with dispase (5 U/ml) (Stemcell Technologies), DNase I (2000 U/ml) (Worthington Biochemical), and SUPERase (1 U/μl) (ThermoFisher Scientific). The tissues were shaken at 350 rpm for 60 min at 37 °C in an Eppendorf Thermomixer R and then titrated with a 200 μl pipette to dissociate cells. Cells were centrifuged at ~ 3000 g for 5 min and suspended in a new solution by titration. Ovomucoid trypsin inhibitors (10 mg/ml) were added to the 2% BSA in HBSS block solution to inhibit proteases. Samples were kept on ice and protected from light for blocking and antibody incubations. Primary antibody solution contained anti-Cd11b, anti-CD45, anti-Cd11c, and DAPI. Samples were blocked for 1 h, incubated with primary antibodies in block solution for 2 h, washed 3×, incubated in secondary antibodies for 1 h, washed 3×, and then suspended in block solution for flow cytometry on BD Biosciences LSR II SORP. Tissue collected from the spleen and processed the same was used to guide analysis of the myeloid cell populations.

### Statistics

Comparisons of mean IOP levels, RGC layer neuron counts, PERG amplitudes, and myeloid cell population numbers were comparisons between *C3ar1*^−/−^ and *C3ar*^+/+^ mice at each age shown and performed using Student’s *t* tests. Each assay involved multiple comparisons and *P* < 0.01 was considered significant. Fisher’s exact test of independence was used to compare the number of nerves at each grade level at a specific age between *C3ar1*^−/−^ and *C3ar*^+/+^ mice. *P* < 0.01 was considered significant. DE genes from RNA sequencing experiments were adjusted for multiple testing using FDR. Genes were considered to be differentially expressed between *C3ar1*^−/−^ and *C3ar1*^*+/+*^ at FDR < 0.01. Ingenuity pathway analysis software was used to assess enrichment of terms (canonical pathways and upstream regulators) by DE genes. Benjamin-Hochberg adjusted *P* values < 0.05 were considered significant. The complete list of genes detected by RNA sequencing was used as the background gene list. Expression data and analyses are provided in Tables S1, S2, and S3.

## Results

### C3ar1-deficient DBA/2J mice developed elevated intraocular pressure similar to C3ar1 sufficient mice

**Fig. 1:**
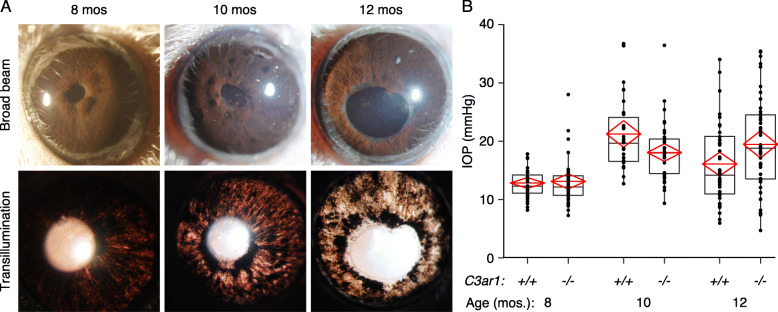
*C3ar1* deficient DBA/2J mice developed iris disease and ocular hypertension similar to *C3ar1* sufficient mice. **a** Regular anterior eye exams were performed using broad beam and transillumination. The pattern of iris depigmentation observed was similar in *C3ar1*^+/+^ (not shown) and *C3ar1*^−/−^ mice (*N* = 80). **b** Elevated IOP compared to young mice was observed in a small subset of mice at 8 months of age and became more prevalent in older mice. No significant difference in IOP was found in *C3ar1*^−/−^ mice compared to *C3ar1*^+/+^ mice at any age (8 mos., *N* = 80, *P* = 0.44; 10 mos., *N* = 80, *P* = 0.23; 12 mos., *N* = 80, *P* = 0.19). Boxes define the 75th and 25th percentiles and their middle line indicates the median value. The diamonds define the 95% confidence interval and their middle line indicates the mean value

DBA/2J mice inherit a depigmenting iris disease that leads to high IOP and glaucoma [33, 39]. Immune cells that are likely to express *C3ar1* contribute to iris damage and the development of ocular hypertension [45, 46]. To determine whether *C3ar1* deficiency affected iris disease or IOP elevation, eyes of *C3ar1*^−/−^ mice and their *C3ar1*^*+/+*^ littermates were examined regularly beginning at 6 months of age. No differences between genotypes were observed in the onset and progression of the iris disease (Fig. 1a) or IOP elevation (Fig. 1b). In *C3ar1*-deficient mice, high IOP sufficient to cause ocular hypertensive damage was observed, similar to standard DBA/2J mice [39].

### C3ar1 deficiency lowered the incidence of glaucomatous degeneration in D2 mice at 10.5 months of age

**Fig. 2:**
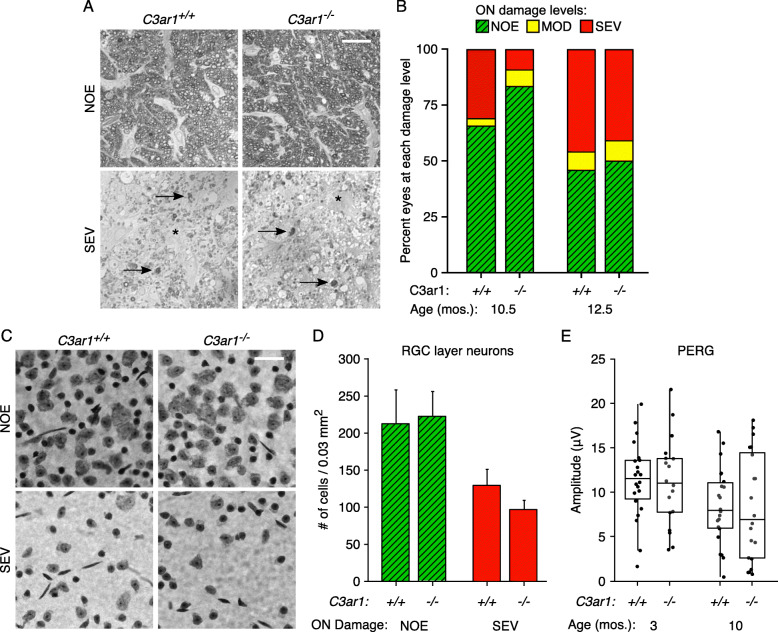
Optic nerve and soma degeneration in *C3ar1*^−/−^ and *C3ar1*^+/+^ mice. **a** Degeneration in PPD-stained optic nerve cross-sections was evaluated based on the presence of axon loss, degenerating axons, and scarring. Examples of degenerating axons (arrow) and glial scarring (asterisk) are indicated. Optic nerve damage in each nerve was classified as ‘no or early’ (NOE), ‘moderate’ (MOD), and ‘severe’ (SEV; see the ‘Methods’ section). Similar signs of degeneration were observed in *C3ar1*^−/−^ and *C3ar1*^+/+^ mice. **b** Distribution of optic nerve damage by genotype and age. At 10.5 months of age (mos.), a significantly lower percentage of eyes in *C3ar1*^−/−^ mice had identifiable glaucomatous degeneration (MOD or SEV damage level; *N* = 110; *P* < 0.0001). At 12.5 mos, there was no longer a difference in the incidence of optic nerve degeneration between genotypes (*N* = 110; *P* = 0.59). **c**, **d** As axonal and somal degeneration of RGCs can be uncoupled by some mutations [40], RGC layer cells were assessed in Nissl stained retinal flat mounts from mice with and without optic nerve degeneration (SEV and NOE, respectively). Genotype had no effect on RGC degeneration in relation to axon loss. The number of RGC layer cells in eyes with healthy optic nerves was similar in *C3ar1*^−/−^ and *C3ar1*^+/+^ mice. Loss of RGC layer cells in eyes with severe optic nerve damage was independent of *C3ar1* genotype. **e** Mean PERG amplitudes were determined in the eyes of young (3 mos.), normotensive and older (10 mos.), ocular hypertensive mice. At 10 months of age, a majority of standard DBA/2J mice do not have significant optic nerve degeneration. *C3ar1* deficiency had no influence on mean PERG amplitude at ages before or after they were affected by ocular hypertension. Boxes define the 75th and 25th percentiles and their middle line indicates the median value. Scale bars: 50 μm

The presence of optic nerve degeneration in an eye can be explicitly determined by identifying degenerating axons and scarred regions with axon loss in the optic nerve (Fig. 2a) [21, 37–40]. The percentage of eyes with optic nerve degeneration in *C3ar1*^−/−^ and *C3ar1*^*+/+*^ mice was compared at 10.5 and 12.5 months of age. At 10.5 months of age, significantly fewer eyes from *C3ar1*^−/−^ mice had degeneration (Fig. 2b), suggesting that *C3ar1* deficiency decreased the risk of ocular hypertensive injury. By 12.5 months of age, *C3ar1*-deficient mice were no longer protected from glaucomatous degeneration (Fig. 2b). Thus, *C3ar1* was not the sole trigger for degeneration, but did promote optic nerve damage.

Eyes from *C3ar1*^−/−^ mice with healthy optic nerves had a normal number of RGC layer neurons, suggesting that *C3ar1* deficiency had not caused abnormal loss of RGCs or amacrine cells (Fig. 2c, d). In eyes with optic nerve degeneration, the loss of RGC layer neurons was independent of *C3ar1* genotype (Fig. 2c, d). The observed loss of approximately half of RGC layer neurons is consistent with cell loss due to optic nerve injury, where the majority of RGCs die and amacrine cells are not affected [37, 40]. These data indicate that *C3ar1*^−/−^ mice had the same type of injury as standard D2 mice.

To investigate changes in RGC function in *C3ar1*^−/−^ mice, pattern electroretinography was used. PERG amplitude is a sensitive measure of RGC activity and detects RGC dysfunction in ocular hypertensive DBA/2J mice [47, 48]. PERG amplitude was recorded at 4 months of age, prior to the elevation of IOP, and 10 months of age, when lower amplitudes are expected due to ocular hypertension and not due to the degeneration that typically occurs at slightly older ages. *C3ar1* deficiency had no effect on the average PERG amplitude in young mice. *C3ar1*^−/−^ mice also had a similar decrease in PERG amplitude due to chronically elevated IOP as *C3ar1*^+/+^ mice. Thus, *C3ar1* deficiency did not prevent changes in RGC activity associated with ocular hypertension (Fig. 2c).

### Ocular hypertension affects C3ar1 expression in the optic nerve head

**Fig. 3:**
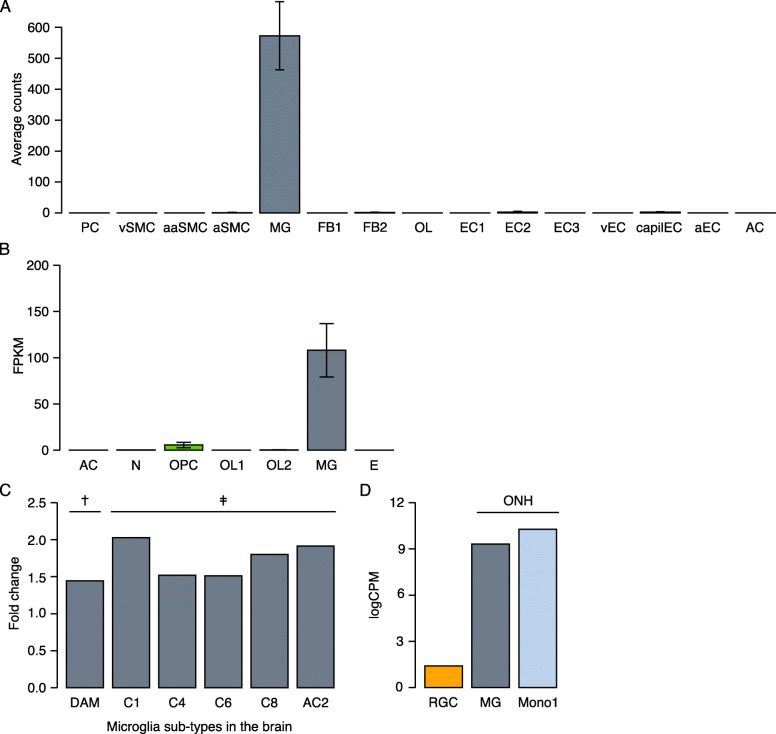
Microglial expression of *C3ar1* in healthy and inflammatory states. **a** Average data from single cell RNA sequencing of healthy brain tissue performed by the Betsholtz laboratory [50, 51] show little to no expression *C3ar1* in cell types other than microglia. **b** Average data from single cell RNA sequencing of brain tissue performed by the Barres laboratory [52] show little to no expression *C3ar1* in cell types other than microglia. **c** Single-cell RNA sequencing data are shown from the laboratories of Amit^†^ [53] and Stevens^‡^ [54]. These studies defined sub-types or clusters of microglia based on differences in gene expression. The relative expression of *C3ar1* in microglia was higher in microglia sub-types associated with macrophage-like activity or inflammation, which are shown here. **d** Expression of *C3ar1* in RNA sequencing data from pooled cells of the indicated cell type sorted from retina or optic nerve tissue from 9 month old DBA/2J mice performed by the John laboratory [55, 56]. Astrocytes (AC), disease-associated microglia for 5xFAD mice (DAM), endothelial cells (EC), endothelial-related cells (E), fibroblast-like (FB), microglia (MG), monocytes (Mono), neurons (N), oligodendrocytes (OL), OL progenitors (OPC), pericytes (PC), smooth muscle cells (SMC), and microglia subtype clusters from Stevens: embryonic microglia (C1), postnatal microglia (C4), *Ms4a7*-positive microglia (C6), *Ccl4*-positive microglia (C8), sub-type in aging mice (AC2)

In DBA/2J mice, observable injury occurs at the optic nerve head (ONH) prior to other regions of the optic nerve [37]. At this same time point, the expression of *C3ar1* increased in the ONH (2.0- to 3.4-fold; *q* < 0.05), but not in the retina (1.0-fold; *q* = 0.85) based on publicly available data [49]. In the healthy brain, it is well established that microglia primarily express *C3ar1*, with low or no expression in other cells (Fig. 3a, b, [50–52]). In addition, higher levels of expression have been observed in subsets of microglia thought to mediate neuroinflammation, such as disease-associated microglia in 5xFAD mice, a widely used mouse model of Alzheimer’s disease and *Ccl3/Ccl4*-positive microglia in aged and white matter-injured brain, as well as at embryonic and postnatal ages of development (Fig. 3c, [53, 54]). This expression pattern is consistent with cell-type specific data from DBA/2J mice. ONH microglia and infiltrating monocytes express *C3ar1* at high levels, while RGCs express *C3ar1* at a lower level (Fig. 3d, [55, 56]). Thus, *C3ar1* deficiency in microglia and monocytes may affect their function or number in the ONH of ocular hypertensive eyes based on these expression data.

### C3ar1 deficiency altered the inflammatory phenotype of cultured microglia

**Fig. 4:**
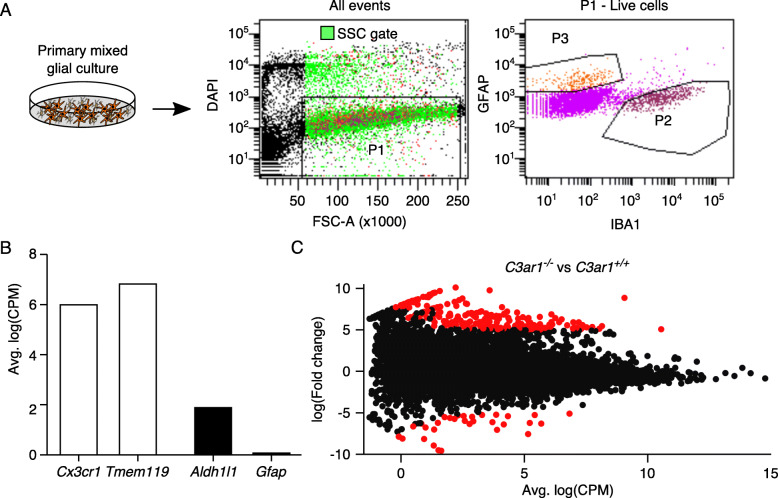
Isolation and RNA sequencing of *C3ar1*^−/−^ and *C3ar1*^*+/+*^ microglia from primary culture. **a** Cells from primary mixed glial cultures were sorted using FACS. A population of microglia was selected for sequencing from live cells (P1) based on high expression of IBA1 and low expression of GFAP (P2). Conversely, a population of astrocytes with high GFAP expression is indicated by P3. **b** The sequenced cells were enriched in microglia based on a high level of expression of microglia genes *Cx3cr1* and *Tmem119* and a low level of astrocyte genes *Aldh1l1* and *Gfap.* **c** Changes in gene expression between *C3ar1*^−/−^ and *C3ar1*^*+/+*^ microglia visualized by MA plot. Points that represent the 408 differentially expressed genes (FDR < 0.005) are colored red

To determine how *C3ar1* deficiency may alter microglia function, RNA sequencing was performed on microglia sorted from primary co-cultures of postnatally derived astrocytes and microglia. In culture, where gene expression is more uniform compared to DBA/2J mice, glial cells express many neuroinflammatory genes expressed in the optic nerve head of DBA/2J mice, including *C3* [18]. Microglia were identified by fluorescence-activated cell sorting as IBA1-positive and GFAP-negative cells (Fig. 4a). The selected cells expressed high levels of genes associated with microglia and low levels of genes associated with astrocytes (Fig. 4b). Four hundred and eight genes were differentially expressed (DE) in microglia due to *C3ar1* deficiency (Fig. 4c; *N* = 6, FDR < 0.005)*.*

**Fig. 5:**
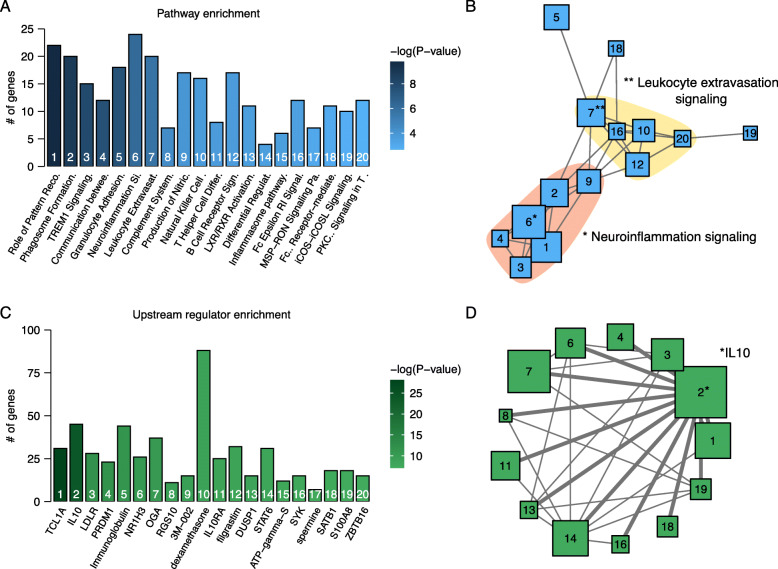
Network analyses identified clusters of changes in neuroinflammation and IL10 signaling pathway gene expression. **a** Top 20 canonical pathways in IPA ranked by *P* value for enrichment in genes differentially expressed between *C3ar1*^−/−^ and *C3ar1*^*+/+*^ microglia. **b** A network of canonical pathways was generated with edges representing that more than 5 genes were shared between two pathways. This network identified that the pathways shown in (**a**) had common biological function related to neuroinflammation (salmon) and immune cell activation (yellow). **c** Top 20 upstream regulators in IPA ranked by *P* value for enrichment in regulating genes differentially expressed between *C3ar1*^−/−^ and *C3ar1*^*+/+*^ microglia. **d** A network of upstream regulators was generated with edges representing that more than 5 genes were shared between two upstream regulators. Only endogenous upstream regulators were included in the network. IL10 had the most connections to other upstream regulators (thick gray edges) and is a potential driver of changes associated with *C3ar1* deficiency

The biological pathways most significantly enriched in DE genes included ‘role of pattern recognition receptors in recognition of bacteria and viruses,’ ‘phagosome formation,’ and ‘TREM1 signaling’ (Fig. 5a). A network of the top 20 enriched pathways, with connections based on having more than five genes in common, suggested that most pathways were closely interrelated and relevant to neuroinflammation and immune cell recruitment (Fig. 5b). Thus, the pathways altered by *C3ar1* deficiency regulate homeostatic and pathological responses in microglia and other immune cells. Upstream regulators of DE genes were analyzed to determine how *C3ar1* deficiency may have this effect (Fig. 5c). The most significantly enriched upstream regulators were ‘TCL1A,’ ‘IL10,’ and ‘LDLR.’ The endogenous regulator that had the highest interconnectivity was the anti-inflammatory cytokine IL10 (Fig. 5d). In addition, the predicted regulator associated with the most DE genes was dexamethasone, a corticosteroid that prevents inflammation. These data show that *C3ar1* deficiency significantly altered the expression of inflammatory genes and signaling pathways in microglia.

### C3ar1 deficiency altered myeloid cell populations in the optic nerve head

**Fig. 6:**
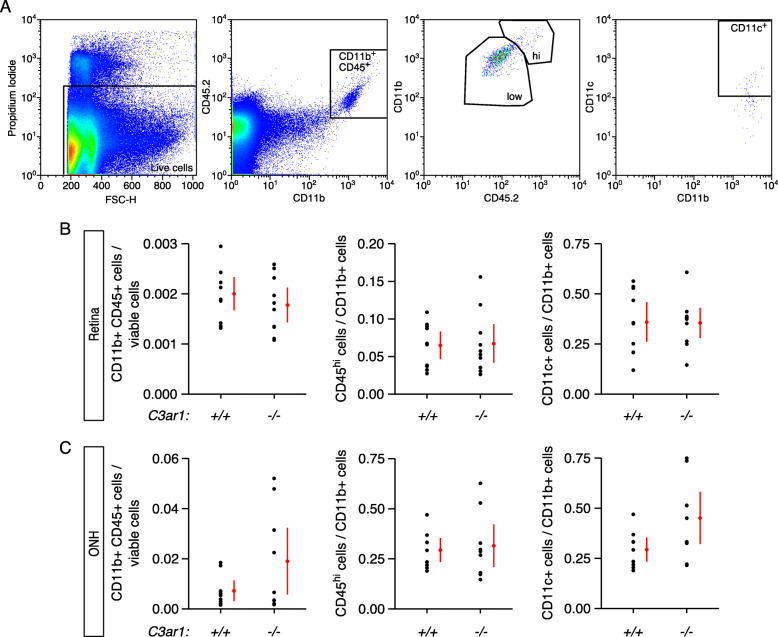
*C3ar1* deficiency altered the population of myeloid-derived cells in the ONH in a subset of eyes. **a** Diagram of the gating strategy used in flow cytometry to identify sub-populations of myeloid-derived cells isolated from retina and optic nerve head tissue. **b** No gross difference in the population profile of these cells was observed in the retinas from ocular hypertensive *C3ar1*^−/−^ and *C3ar1*^*+/+*^ mice. **c** The number of myeloid cells detected in optic nerve head tissue was more variable *C3ar1*^−/−^ mice than in *C3ar1*^+/+^ mice

In *C3ar1*-deficient DBA/2J mice, changes in inflammatory gene expression are likely to change the localization or reactivity of microglia and monocytes. To investigate this in DBA/2J mice, the population of myeloid-derived cells in the retina and the optic nerve head was assessed by flow cytometry at 10 months of age (Fig. 6a). In the retina, no difference was observed between *C3ar1*^+/+^ and *C3ar1*^−/−^ mice in the percentage of myeloid-derived cells, including CD45^hi^ and Cd11c^+^ monocytes (Fig. 6b). Thus, *C3ar1* deficiency did not have a general effect on the number of these cells in neural tissue exposed to ocular hypertension. In contrast to the retina, the ONH is a very small region of tissue more sensitive to ocular hypertensive stress and a location where myeloid cells likely have beneficial and harmful effects at different stages of disease [21, 55, 57]. In the ONH of *C3ar1*^−/−^ mice, the number of myeloid cells was more variable compared to in *C3ar1*^*+/+*^ mice (Fig. 6c). These data suggest a role for *C3ar1* in regulating myeloid cells in ONH under chronic ocular hypertensive stress.

## Discussion

Interventions that target complement activation are being evaluated in many types of neurological injury and disease (reviewed in [58]). DBA/2J mice are a useful model for testing whether neurodegeneration caused by chronic ocular hypertension is prevented by targeting specific components of the complement cascade. DBA/2J mice have an inborn deficiency in C5 that prevents secretion of C5 and formation of both C5a and the MAC (which requires C5b). Therefore, optic nerve damage in these mice is independent of secreted C5, which has been shown to be detrimental if present [22]. However, optic nerve damage in DBA/2J mice is still dependent on C1q based on the protection against ocular hypertension observed in *C1qa*^−/−^ mice [38]. To determine how C1q causes permanent damage and vision loss, there are a limited number of targets remaining to investigate, such as C3, C4, and receptors for C1q. As shown here, C3a contributes to degeneration caused by ocular hypertension based on the decreased incidence of optic nerve damage at 10.5 months of age in *C3ar1*^−/−^ mice.

Understanding why *C3ar1* deficiency did not provide long-lasting protection requires understanding other damaging consequences of complement activation. Greater protection in DBA/2J mice has been achieved by disrupting *C1qa* [38] compared to *C3ar1*, suggesting that *C1qa* triggers multiple damaging responses. A therapy targeting sites opsonized by C3b and C4b, achieved by expression of CR2-Crry in retinal ganglion cells, has produced results more similar to *C1qa* deficiency [15]. Crry would be predicted to inhibit C3 convertase activity of the classical pathway (through C4b) and alternative pathway (through C3b) [59], severely limiting accumulation of both C3a and C3b in the treated DBA/2J mice. The results of treatment with CR2-Crry suggest that inhibition of C3a and C3b may protect in an additive manner. In fact, DBA/2J mice that lack the C3b receptor CR3, by disruption of *Itgam*, are less vulnerable to optic nerve degeneration [55]. Similar to *C3ar1*^−/−^ mice, *Itgam*^−/−^ mice are not protected as well as *C1qa*^−/−^ and CR2-Crry treated mice. These results suggest that complement activated peptides and opsonization products may independently contribute to optic nerve degeneration. Thus, targeting both types of receptors, such as by disrupting both *C3ar1* and *Itgam* expression, may protect to a greater degree than targeting *C3ar1* or *Itgam* alone and explain the full effect of treatment with CR2-Crry or disrupting *C1q*.

Complement activation is primarily expected to guide a targeted immune cell response in DBA/2J mice, given the deficiency of secreted C5 and lack of MAC activation. A type of targeted response to C1q and C3a by microglia and other myeloid cells is to phagocytose neuronal blebs or dying neurons and limiting pro-inflammatory cytokine production [60–62]. C1q regulates dendritic and synaptic pruning during development and ocular hypertension in the retina [23, 63]. *C3ar1* has been implicated in mediating synaptic plasticity [26] and phagocytosis by microglia [64] in cell culture and a mouse model relevant to Alzheimer’s disease, but has not yet been in models of glaucoma. In this study, C3ar1 deficiency did not influence PERG readings that occur in conjunction with synapse loss and dendritic remodeling. It is possible that C3AR1 signaling does not strongly affect phagocytosis or synapse loss in an ocular hypertensive setting and that C3AR1 signaling has detrimental effects in glaucoma through a different mechanism.

To predict how *C3ar1* deficiency might affect microglial cell biology, cell culture of microglia was used as a model to identify differences in gene expression caused by *C3ar1* deficiency. Numerous genes associated with inflammation were affected by *C3ar1* expression raising the possibility that the effect of disrupting *C3ar1* on neurodegeneration may be caused by a change in inflammation. More specifically, a significant number of changes in gene expression were associated with downstream effects of IL10 signaling. These data predict crosstalk between C3AR1 and IL10 in microglia. An interaction between C3AR1 and IL10 has been shown previously in another type of immune cell; C3AR1 inhibited IL10 production by CD8^+^ tumor-infiltrating lymphocytes [65]. Interestingly, microglia can produce IL10 and autocrine signaling by IL10 has been suggested to regulate microglial activation [66]. However, it has not been determined whether C3AR1 has an effect on IL10 production or the expression of related genes and proteins in microglia in an ocular hypertensive setting. Furthermore, little is known about whether IL10 signaling is activated or has a function in glaucomatous neurodegeneration. Addressing these questions will help resolve whether *C3ar1* deficiency altered disease risk by modulating inflammation or through a different mechanism.

C3a may also recruit monocytes that express *C3ar1*. A subclass of monocytes (CD11b-positive, CD45-hi, and Cd11c-positive) that express *C3ar1* increase in number in tissue affected by ocular hypertension [21], but how they are recruited is not known. C3a may influence their recruitment based on flow cytometry data presented here, although this is unresolved. In some eyes from *C3ar1*^−/−^ mice, the number of myeloid cells in the optic nerve head appeared to be increased as observed by flow cytometry. It is possible that myeloid cells have a protective role early in disease and that this increase helped prevent optic nerve damage. In this study, it was not feasible to address these possibilities in more depth due to the spontaneous nature of IOP elevation, variability between eyes, and the unexpected increase in myeloid cell population variability in the ONH of *C3ar1*^−/−^ mice. A larger study using DBA/2J mice or another model with chronic ocular hypertension could address how *C3ar1* alters microglia and monocyte localization and function in this type of glaucoma. All of the hypotheses are consistent with the idea that targeting myeloid cells with therapy may improve disease outcomes in glaucoma.

In DBA/2J mice, ONH astrocytes express C3 [18], a marker associated with a neurotoxic phenotype in some conditions [67]. However, *C3* deficiency was shown to increase vulnerability of the optic nerve to ocular hypertensive damage [18]. This is counterintuitive to harmful effects of C3a and C3b and was suggested to implicate early protective responses by astrocytes in glaucoma. The role of C3 in neuroprotective and neurotoxic functions of astrocytes needs to be determined. Astrocytes in DBA/2J mice may be capable of both neuroprotective and neurotoxic function that depends on the activation of specific extracellular receptors. In this case, *C3ar1* deficiency may protect by decreasing the extracellular signals produced by microglia and infiltrating monocytes, including C1Q, IL1A, and TNF [67], that trigger a neurotoxic response. Testing the function of C1q receptors and C3 in astrocytes in DBA/2J mice could better define the effects of complement activation and show whether astrocytes directly contribute to optic nerve degeneration.

## Conclusion

Signaling through C3AR1 promoted neurodegenerative processes in a model of glaucoma with chronic ocular hypertension and neuroinflammation. *C3ar1* deficiency caused changes to IL10-related signaling pathways in cultured microglia, pathways predicted to have an important effect on microglia reactivity. In this regard, genetic and other factors that influence expression of *C3ar1*, *C3*, or other members of the complement cascade may predispose people to beneficial or harmful neuroinflammatory responses by affecting microglial or astrocytic reactivity. Targeting myeloid cells and complement-mediated inflammation pathways with therapies will likely be a beneficial addition to neuroprotective therapeutic strategies by reducing the impact of harmful inflammatory processes.

## Supplementary Information

**Additional file 1:** **Table S1.** DE genes

**Additional file 2:** **Table S2.** Canonical pathway

**Additional file 3:** **Table S3.** Upstream regulators

## Data Availability

The datasets during and/or analyzed during the current study available from the corresponding author on reasonable request.

## Abbreviations

C1q: Complement component 1q complex
C3ar1: C3a receptor
DE: Differentially expressed
IOP: Intraocular pressure
IPA: Ingenuity Pathway Analysis software
NOE: No or early, no readily detectible axon loss
ONH: Optic nerve head
MAC: Membrane attack complex
MOD: Moderate, less than 50% of axons damaged/lost,
PERG: Pattern electroretinography
PPD: Paraphenylenediamine
SEV: Severe, more than 50% of axons damaged/lost
